# Literary Notes

**Published:** 1909-06-12

**Authors:** 


### LITERARY NOTES.

The National Food Reform Association, whose temporary address is 40 Chandos Street, Charing Cross, London, have just published two booklets. The first, by the Chairman, the Hon. Neville Lytton, deals with the need for food reform at the present day and the aims of the Association; while the second, entitled "Hints towards Diet Reform," with 24 simple recipes, will be found most useful by all who desire to introduce greater variety into their menus. Specimen copies will be sent post free by the Secretary on the receipt of three stamps.

Messrs. John Bale, Sons and Danielsson, Ltd., are publishing a translation of the second edition of Bandelier and Roepke's "Lehrbuch der specifischen Diagnostik und Therapie der Tuberkulose" under the title of "Tuberculin in Diagnosis and Treatment." The first section (eighty pages) deals with the subcutaneous tuberculin test and the local tests associated with the names of Calmette and v. Pirquet, discussing their significance in diagnosis. The second section (seventy-five pages) describes the treatment of pulmonary tuberculosis with tuberculin, giving the exact mode of employment of the different preparations. The third section (twelve pages) summarises the tuberculin treatment of other organs. A coloured plate of the local tests is given, and eighteen charts illustrating the tuberculin reaction and the course of tuberculin treatment.

All concerned in the physical conditions and education of the children of the poor will be interested in a new work by A. D. Edwards, M.B., B.S., B.Sc., D.P.H., Medical Officer of Schools for Bournemouth, entitled "Children of of Poor." The book deals with such subjects as "Child-Life in the Slums," "Underfed and Overworked Children," "Pain and Suffering in Childhood," "Childhood in the Workhouse," "Open-air Schools," "The Children Act of 1908," etc., and is an indictment of the unsatisfactory conditions under which poor children are reared and educated, and of the avoidable unhappiness and pain which they suffer. Remedial measures are fully dealt with. The publishers are Hammond, Hammond and Company, 12 Paternoster Row, E.C.

Acting in pursuance of a resolution passed at the last International Tuberculosis Conference, the International Anti-Tuberculosis Association have sent out from their office in Berlinerstrasse, Charlottenburg, an inquiry sheet relating to the details of expenditure in sanatoriums and kindred institutions. The questions, although not expressed in very idiomatic English (the word "surrogate" used in connection with butter suggests a good many things sooner than margarine, for instance), are comprehensive and practical, and, if well answered, should certainly put the committee appointed to deal with this matter in possession of some valuable information. There can be no doubt that any measure, however slight, of central control is likely to benefit sanatoriums just as much as hospitals. It may be noted that the schedule, while inquiring as to means of support, makes no specific mention of work done by patients.

